# Outcomes of direct-acting antivirals in patients with HCV decompensated cirrhosis: a systematic review and meta-analysis

**DOI:** 10.3389/fmed.2023.1295857

**Published:** 2023-11-29

**Authors:** Tanawat Jongraksak, Alan Chuncharunee, Pongphob Intaraprasong, Amarit Tansawet, Ammarin Thakkinstian, Abhasnee Sobhonslidsuk

**Affiliations:** ^1^Division of Gastroenterology and Hepatology, Department of Medicine, Ramathibodi Hospital, Mahidol University, Bangkok, Thailand; ^2^Department of Clinical Epidemiology and Biostatistics, Mahidol University, Bangkok, Thailand; ^3^Department of Surgery, Faculty of Medicine Vajira Hospital, Navamindradhiraj University, Bangkok, Thailand

**Keywords:** hepatitis C virus, decompensated cirrhosis, direct-acting antiviral agent, overall survival, hepatocellular carcinoma, MELD score

## Abstract

**Background:**

Direct-acting antivirals (DAA) are effective for chronic hepatitis C virus (HCV) treatment. However, their impact on overall survival (OS), hepatocellular carcinoma (HCC) occurrence, HCC-free survival, and liver function in patients with HCV decompensated cirrhosis remains uncertain. This study aimed to evaluate the effects of DAA treatment on this population.

**Methods:**

Studies were identified by searching the MEDLINE, SCOPUS, and CENTRAL databases. OS and HCC-free survival probabilities and time data were extracted from Kaplan-Meier curves. A one-stage meta-analysis using parametric Weibull regression was conducted to estimate the relative treatment effects of DAA vs. no DAA. The primary outcome was the OS rate. The secondary outcomes were HCC-free survival, HCC occurrence rate, and improvement in the Model for End-stage Liver Disease (MELD) score.

**Results:**

Eight cohorts comprising 3,430 participants (2,603 in the DAA group and 1,999 in the no-DAA group) were included. The OS probabilities at 12 and 24 months were 95 and 90% for the DAA group, respectively, compared with 89 and 80% in the no-DAA group, respectively. Hazard ratio (HR) was 0.48 (95% confidence interval (CI): 0.39, 0.60; *p* < 0.001). The HCC-free survival probabilities at 12 and 24 months were 96 and 90%, respectively, in the former, and 94 and 85%, respectively, in the latter. The HR of HCC occurrence was 0.72 (95% CI: 0.52, 1.00; *p* = 0.05), which suggests that DAA treatment in decompensated cirrhosis may lead to a 28% lower risk of HCC occurrence. The mean MELD score difference was −7.75 (95% CI: −14.52, −0.98; *p* = 0.02).

**Conclusion:**

Improvement in OS and MELD score is a long-term benefit of DAA treatment in patients with HCV decompensated cirrhosis, with a marginal effect of the treatment on HCC development.

## 1. Introduction

Hepatitis C virus (HCV) infection is a common cause of chronic viral hepatitis and end-stage liver disease, worldwide (1). According to the World Health Organization (WHO), approximately 58 million individuals are infected with HCV (2). Chronic HCV infection can progress to decompensated cirrhosis, resulting in significant morbidity and mortality, and the need for liver transplantation (3). Interferon-based therapies are unsuitable for patients with decompensated cirrhosis owing to their severe adverse effects (4). However, with the advent of interferon-free direct-acting antiviral (DAA) agents (5, 6) the therapeutic landscape for HCV infection has dramatically changed. These new agents offer higher sustained virological response (SVR) rates and can effectively eradicate HCV infection (7). Treatment regimens consisting of 12 or 24 weeks of DAAs, with or without ribavirin, have become the cornerstone of chronic HCV treatment (5, 8).

Although the European Association for the Study of the Liver (EASL) guidelines recently recommended the use of DAAs for the treatment of decompensated HCV cirrhosis, the clinical efficacy and safety of these agents in this context have not yet been fully confirmed (9). DAA treatment in decompensated cirrhosis has shown promising results in terms of high SVR rates, although improvements in liver function tests and overall survival (OS) have been limited (10–12). However, a recent study has demonstrated a reduction in both all-cause and liver-related mortality following DAA treatment in patients with decompensated cirrhosis (12, 13). Despite these findings, the long-term benefits of HCV eradication in this patient population warrants further investigation (13).

This systematic review and meta-analysis aimed to assess long-term clinical outcomes such as OS, occurrence of hepatocellular carcinoma (HCC), HCC-free survival, and improvement of liver function, which was defined as a decline in the Model for End-stage Liver Disease (MELD) score in patients with decompensated HCV cirrhosis who received DAA treatment.

## 2. Materials and methods

The entire process of this systematic review and meta-analysis was conducted in accordance with the Preferred Reporting Items for Systematic Reviews and Meta-Analyses (PRISMA) statement (14). Additionally, the study protocol was registered in PROSPERO for systematic reviews (registration number: CRD42022316276).

### 2.1. Data sources and searches

Two investigators (TJ and AS) independently conducted a systematic search for relevant studies in MEDLINE, SCOPUS, and the Cochrane Central Register of Controlled Trials (CENTRAL) from their inception to June 30, 2023. The following search strategy was used: “chronic hepatitis C” OR “hepatitis C” AND “hepatic cirrhosis” OR “decompensated cirrhosis” OR “advanced cirrhosis” OR “end-stage liver disease” AND “direct-acting antiviral” OR “sofosbuvir” OR “DAA” OR “SOF” AND “mortality” OR “death” OR “survival” OR “prognosis” OR “hepatocellular carcinoma” OR “hepatocellular carcinoma occurrence” OR “HCC” OR “liver function test” OR “Model for End-stage Liver Disease” OR “Child-Pugh”(Table 1). Additionally, the reference lists of all the retrieved review articles were examined to identify potentially relevant articles that may have been missed by the search system (15).

**TABLE 1:** Searching method.

Domains	Search term	Search strategy
P1-patient1	• “Chronic hepatitis c”	#1
	• “Hepatitis c”	#2
	• HCV	#3
All P1	#4	#1 OR #2 OR #3
P2-patient2	• “Hepatic cirrhosis”	#5
	• “Decompensated cirrhosis”	#6
	• “Advanced cirrhosis”	#7
	• “End stage liver disease”	#8
All P2	#9	#5 OR #6 OR #7 OR #8
All P	#10	#4 AND #9
E-exposure	• “Direct acting antiviral”	#11
(I-intervention	• DAA*	#12
/C-comparator)	• Sofosbuvir	#13
	• SOF	#14
All E	#15	#11 OR #12 OR #13 OR #14
O-outcome	• Mortality	#16
	• Death	#17
	• Survival	#18
	• Prognosis	#19
	• “Hepatocellular carcinoma”	#20
	• HCC	#21
	• “Hepatocellular carcinoma occurrence”	#22
	• “Liver function test”	#23
	• “Child Pugh”	#24
	• Model for end stage liver disease”	#25
	• MELD	#26
All-O	#27	#16 OR #17 OR #18 OR #19 OR #20 OR #21 OR #22 OR #23 OR #24 OR #25 OR #26
Over all (P1 AND P2 AND E AND O)	#28	#10 AND #15 AND #27
	((Chronic hepatitis c[MeSH Terms]) OR (Hepatitis c[MeSH Terms]) OR (HCV)) AND ((Hepatic cirrhosis[MeSH Terms]) OR (“Decompensated cirrhosis”) OR (“Advanced cirrhosis”) OR (“End stage liver disease”)) AND ((Direct acting antiviral) OR (Sofosbuvir[MeSH Terms]) OR (DAA*) OR (SOF)) AND ((Mortality) OR (death) OR (survival) OR (Prognosis) OR (hepatocellular carcinoma[MeSH Terms]) OR (“hepatocellular carcinoma occurrence”) OR (HCC) OR (“Liver function test”) OR (MELD) OR (“Model for end stage liver disease”) OR (“Child Pugh”))	

### 2.2. Study selection

Two independent reviewers (TJ and AS) assessed the eligibility of each article by screening titles and abstracts. Full articles were obtained for comprehensive evaluation of cases in which eligibility was unclear. The kappa statistic was used to measure agreement between the two reviewers. In instances of discordant decisions between the two investigators, the articles were subjected to a full-text review. Any discrepancies were resolved through consensus between the two reviewers with the involvement of a third investigator (AT) when necessary.

### 2.3. Selection criteria

Studies were considered eligible if they fulfilled the following criteria: (1) they had study designs of randomized controlled trials (RCTs), prospective or retrospective cohorts with a sample size greater than 10; (2) they included adult patients aged 18 years and older with decompensated HCV cirrhosis; (3) they included any interferon-free DAA therapy as the treatment group compared with a control group receiving only the standard of care for decompensated cirrhosis; and (4) they reported one or more of the following outcomes: OS, HCC occurrence, liver function tests (LFTs), MELD score, or Child-Pugh score after treatment. The following studies were excluded: (1) duplicated studies; (2) studies involving post-liver transplant patients; (3) studies with insufficient data; and (4) studies in which the authors could not provide the full unpublished article after two separate contact attempts spaced 1 month apart.

### 2.4. Outcome measures

The primary outcome of this study was OS of patients with decompensated HCV cirrhosis. Secondary outcomes included the occurrence rate of HCC, HCC-free survival, and improvement in liver function, which was defined as a decline in the MELD score. The MELD score was calculated using the following formula: 3.78 × ln[serum bilirubin (mg/dL)] + 11.2 × ln[INR] + 9.57 × ln[serum creatinine (mg/dL)] + 6.43 (16). The MELD scores range from 6 to 40, with higher scores indicating a higher 3-month mortality risk related to liver disease (17).

### 2.5. Data extraction and quality assessment

Data extraction from the included studies was performed independently by two investigators (TJ and AS). The extracted data included the first author’s name, year of study, country of study population, demographic information, baseline LFT results, and MELD score. Summary statistics, such as hazard ratios (HR) for HCC occurrence and 1-year survival rates were also collected. In cases where these summary statistics were not provided, the GetData Graph Digitizer program version 2.26.0.2017 (18) was used to extract data from Kaplan–Meier curves. Subsequently, Kaplan–Meier data were transformed into individual patient data (IPD) using the “ipdfc” command in STATA 17. Any missing data were sought by contacting the authors of the respective studies via e-mails.

The quality of the included studies was assessed using the Cochrane Risk of Bias in Non-randomized Studies of Interventions (ROBINS-I) tool (19). This tool evaluates seven domains: confounding, study selection, classification of interventions, deviations from intended interventions, missing data, outcome measurements, and selection of reported results. Studies were categorized as having a high risk of bias if at least one domain had a high risk of bias. Conversely, studies were considered to have a low risk of bias if all the domains had a low risk of bias. If some domains had a low risk of bias and others had concerns, the studies were classified as having a moderate risk of bias.

### 2.6. Statistical analysis

Overall survival and HCC-free survival probabilities and time data were extracted from the Kaplan–Meier curves. A one-stage meta-analysis was conducted using multilevel parametric survival regression with a Weibull distribution (20) to compare the relative treatment effects of DAA compared to the control (no DAA) group for the IPD analysis of overall and HCC-free survival and an improvement in the MELD score. The median survival time for each treatment group and HRs with their corresponding 95% confidence intervals (CIs) (21) were estimated accordingly. For summary statistical data, a meta-analysis was performed using a random-effects model if heterogeneity was present, as indicated by Cochran’s *Q*-test *p*-value < 0.1, or an I-square (*I*^2^) statistic >25%. A fixed effects model was used in the absence of heterogeneity. Publication bias was assessed using funnel plots and Egger’s test. All statistical analyses were carried out using STATA version 17.0, and a *p*-value < 0.05 was considered statistically significant.

## 3. Results

### 3.1. Search results and study characteristics

**FIGURE 1:**
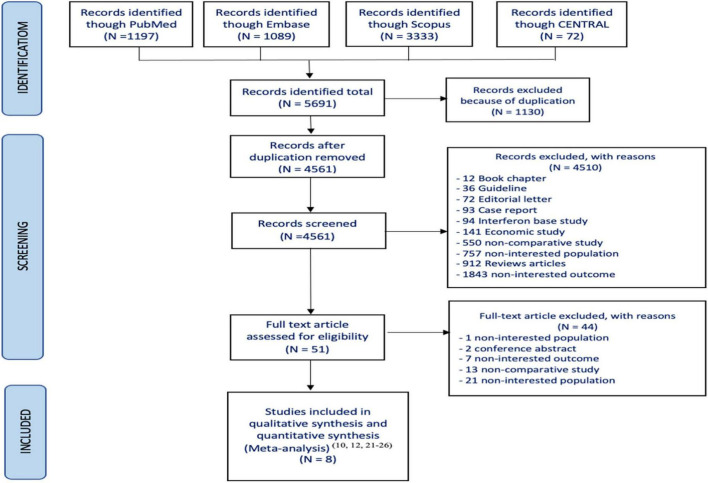
Flowchart of study selection for the systematic review and meta-analysis.

Of the 5,691 articles initially identified, 51 were reviewed for eligibility. However, only 8 studies met the inclusion criteria (10, 13, 22–27) and were included in the systematic review (Figure 1). Among them, five studies reported OS (10, 13, 24, 25, 27) four studies reported HCC occurrence (10, 13, 26, 27) and three reported MELD scores (13, 22, 23). The characteristics of the included studies are summarized in Table 2. All studies had cohort designs, with sample sizes ranging from 150 to 1,093. Eight studies (10, 13, 22–27) which included seven prospective observation cohort studies (10, 13, 22, 23, 25–27) and one retrospective cohort study (24) were enrolled. The mean age of participants ranged from 51 to 59 years, with a mean MELD score of approximately 13. The follow-up time varied from 6 to 40 months, and all studies used interferon-free DAAs regimens with sofosbuvir-based treatments.

**TABLE 2:** Characteristics of the observational studies introduced in the meta-analysis.

References	Country	Study type	Exposure and comparator	Population (DAA/control)	SVR (%)	Follow-up* (month)	Outcomes	Study conclusion(s)
Cheung et al. (10)	United Kingdom	Prospective Cohort	SOF/LDV ±RBV SOF/DCV ±RBV vs. Conservative	406/261	78.1	15	All-cause mortality; HCC occurrence	DAA led to improvement of liver function with no adverse impact nor increase in HCC.
Foster et al. (22)	United Kingdom	Prospective Cohort	SOF/LDV ±RBV SOF/DCV ±RBV vs. Conservative	409/319	NA	6	SVR, adverse outcomes	SVR was associated with improvement in liver function
Essa et al. (23)	Egypt	Prospective cohort	SOF/LDV ±RBV SOF/DCV ±RBV vs. conservative	75/75	NA	6	MELD	Improvements in liver function and QOL
Dellay et al. (24)	United States	Retrospective cohort	Any DAA vs. conservative	154/939	NA	23	Mortality on waiting list, need for LT	No difference in all causes mortality between DAA treated and non-treated groups. DAA use was associated with lower incidence of LT and a trend toward more waitlist removals
Hanafry et al. (25)	Egypt	Prospective cohort	Any DAA vs. conservative	160/80	90.0	24–31	Efficacy and safety	DAA in DC yielded a 90%SVR with improved CTP and MELD
Lusivika-Nzinga et al. (26)	France	Prospective cohort	Any DAA vs. conservative	552/NA	93	36.8	HCC occurrence	A decrease in the risk occurrence of HCC
Kumada et al. (13)	Japan	Prospective cohort	Any DAA vs. conservative	364/249	86.2	36	Liver and non-liver related mortality	SVR by DAA for DC reduced liver and non-liver related mortality
Pageaux et al. (27)	France	Prospective cohort	Any DAA vs. conservative	483/76	88	39.7	All-cause mortality, HCC occurrence, LT, SVR	DAA reduced risk of mortality with SVR of 88%

DAA, direct-acting antivirals; SVR, sustained virological response; HCC, hepatocellular carcinoma; SOF, sofosbuvir; LDV, ledipasvir; DCV, daclatasvir; RBV, ribavirin; MELD, model for end-stage liver disease; QOL, quality of life; LT, liver transplant; CTP, child-turcotte-pugh score; NA, not applicable.

*Median or range.

The risk of bias assessment is reported in Table 3, which shows that all studies had a moderate risk of bias. All included studies were cohort studies and reported a moderate risk of bias in various domains, including confounding, intervention classification, deviation from intervention, measurement of outcomes, and selection of reported results.

**TABLE 3:** Risk of bias assessment for individual studies (non-randomized trials).

References	Confoun-ding	Selection	Intervention classification	Deviation from intervention	Missing data	Measurement of outcome	Selection of reported result	Overall
Cheung et al. (10)	Moderate	Low	Moderate	Moderate	Low	Moderate	Moderate	Moderate
Foster et al. (22)	Moderate	Low	Moderate	Moderate	Low	Moderate	Moderate	Moderate
Essa et al. (23)	Moderate	Low	Moderate	Moderate	Low	Moderate	Moderate	Moderate
Dellay et al. (24)	Moderate	Low	Moderate	Moderate	Low	Moderate	Moderate	Moderate
Hanafry et al. (25)	Moderate	Low	Moderate	Moderate	Low	Moderate	Moderate	Moderate
Lusivika-Nzinga et al. (26)	Moderate	Low	Moderate	Moderate	Low	Moderate	Moderate	Moderate

### 3.2. Overall survival

**FIGURE 2:**
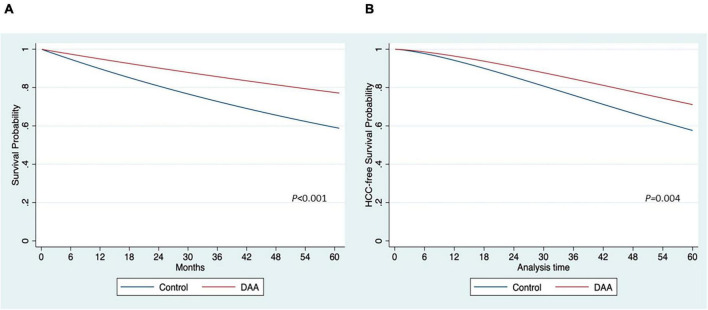
Estimation of survival probability by the treatment of direct-acting antivirals (DAA). **(A)** Overall survival **(B)** hepatocellular carcinoma (HCC)-free survival.

Five studies (10, 13, 24, 25, 27) reported Kaplan–Meier curves for OS data, allowing the generation of IPD by treatment groups. A one-stage meta-analysis using mixed-effect Weibull regression was employed to analyze the OS in the treatment groups (20). Treatment-specific survival probability curves were constructed (Figure 2). The DAA group exhibited a higher OS than the control group (*p* < 0.001) (Figure 2A). At 12, 18, and 24 months, the survival rates in the DAA group were 95, 92, and 90%, respectively. In contrast, the corresponding survival probabilities in the control group were 89, 94, and 80%, respectively. The relative treatment effect was estimated with an HR of 0.48 (95% CI: 0.39, 0.60), indicating that patients who received DAAs had a significantly lower risk of death (52% lower) than those who did not. A mixed-effect Weibull regression model was applied, which indicated that the DAA group had a higher probability of HCC-free survival than did the control group. At 12 and 24 months, the HCC-free survival probabilities were 96 and 90% for the DAA group, and 94 and 85% for the control group, respectively (Figure 2B).

**FIGURE 3:**
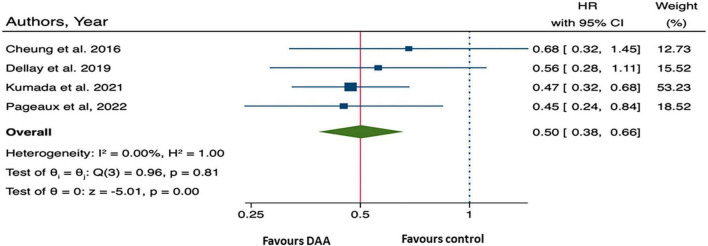
Forest plot of pooling hazard ratios across studies: overall mortality.

Furthermore, the pooled HR from four studies provided summary statistical data of overall mortality was 0.50 (95% CI: 0.38, 0.66), (10, 13, 25, 27) (Figure 3), suggesting that patients who received DAA treatment had a 50% significantly lower risk of all-cause mortality than those who did not.

### 3.3. HCC occurrence

**FIGURE 4:**
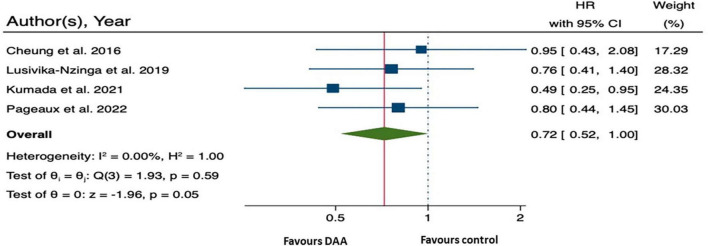
Forest plot of pooling hazard ratios across studies: hepatocellular carcinoma (HCC) occurrence.

Four studies involving 1,832 patients with decompensated HCV cirrhosis were included in the analysis of HCC-free survival following DAA treatment (10, 13, 26, 27). The relative treatment effect or rate of HCC occurrence, as measured by HRs with 95% CI, was 0.72 (0.52, 1.00; *p* = 0.05) (Figure 4). This suggests that patients with decompensated HCV cirrhosis who received DAA treatment may had a 28% lower risk of HCC than the control patients.

### 3.4. MELD score

**FIGURE 5:**
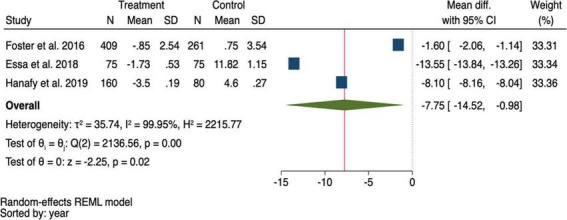
Pooling mean difference of the Model for end-stage liver disease (MELD) score between the direct-acting antivirals (DAA) and control group.

Three studies reported the mean MELD scores after treatment at different time points, ranging from 3 to 6 months (13, 22, 23). When pooling the mean differences in MELD scores, it was found that the MELD score was significantly lower in the DAA group than in the control group, with a mean difference of −7.75 (95% CI: −14.52, −0.98; *p* = 0.020) (Figure 5).

### 3.5. Heterogeneity and publication bias

**FIGURE 6:**
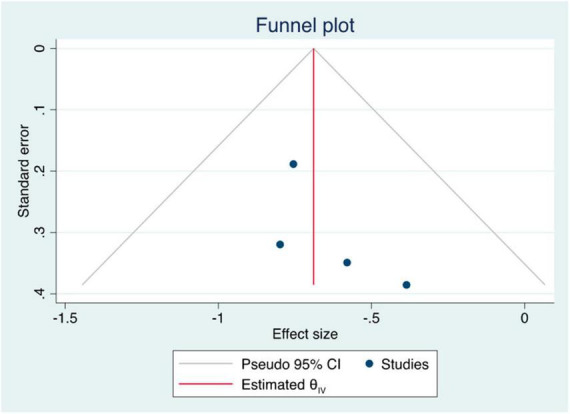
Funnel plot of overall mortality.

The summary data of the HRs of overall mortality and HCC occurrence revealed no heterogeneity (*I*^2^ = 0%) (Figures 3, 4). The mean differences in MELD scores were highly variable across the three studies (13, 22, 23) with an *I*^2^ value of 99.95%, indicating substantial heterogeneity (Figure 5). Funnel plots and Egger’s test of the pooled analysis of overall mortality revealed no evidence of publication bias (Egger’s test, *p* = 0.69) (Figure 6).

## 4. Discussion

We conducted a systematic review and meta-analysis based on data from eight cohorts (10, 13, 22–27) to evaluate the relative treatment effect of DAA compared with the no-DAA approach. This study was directly compared between patients who received DAA and not received DAA, which meant those who received DAA regardless of SVR achievement were classified into DAA group. Our findings revealed that DAA treatment is associated with improved OS and HCC-free survival. At 24 months, the OS and HCC-free survival probabilities were 90 and 90%, respectively, in the DAA treatment groups and 80 and 85%, respectively, in the standard treatment groups. Patients who received DAAs had a significantly lower risk of death (approximately 50% lower) and lower risk of HCC development (28% lower) than those who did not receive DAAs. Additionally, DAA treatment resulted in a notable improvement in liver function, as demonstrated by a reduction of seven points in the MELD scores. Our study contributes valuable information beyond previous studies (5, 12, 28, 29) that mainly reported SVR rates after DAA treatment. We provided additional clinically relevant insights, including survival rates, HCC-free survival, HCC occurrence rates, and MELD score improvement. The included studies primarily included patients with HCV-related decompensated cirrhosis (10, 12, 21–26).

Our study showed a significant improvement in the MELD score following DAA treatment compared with non-DAA or standard treatment. This finding aligns with that of previous studies (9, 23). However, it is important to note that the pooled post-treatment MELD scores exhibited high heterogeneity due to variations in baseline MELD scores across different studies (13, 22, 23). The results might have shown even greater improvement if additional data on the updated MELD scores were available. Nevertheless, our findings suggest that DAA treatment may contribute to ameliorating disease severity in patients with HCV-related decompensated cirrhosis waiting for liver transplantation. Despite the current guidelines recommending DAA treatment for all patients (9) the outcomes of DAA treatment in decompensated cirrhosis remain unclear, mainly because decompensated cirrhosis is often considered a point of no return (30). However, the findings of this meta-analysis confirmed the benefits of DAA treatment in patients with HCV-related decompensated cirrhosis, highlighting the need for further research that may include a cost-effective analysis of DAAs in this population.

To the best of our knowledge, this study is the first systematic review and meta-analysis to compare the long-term clinical outcomes (OS, HCC-free survival, HCC development, and MELD scores) of DAA treatment with those of no DAA treatment in patients with HCV-related decompensated cirrhosis. However, our study has certain limitations. First, the absence of available RCTs limited our analysis to cohort studies, which inherently carries the risk of confounding and selection bias. This limitation is primarily due to ethical considerations as DAA treatment has become the recommended standard therapy for chronic HCV infections. Second, we could not analyze other outcomes, such as improvement in the Child-Pugh score, fibrosis score, and adverse events, because of the limited number of studies reporting these specific outcomes. To address these limitations, future studies should include large-scale cohort studies that incorporate propensity score analysis. Third, because of the limited number of studies available for each outcome of interest, we could not perform a subgroup analysis based on variables such as the Child-Pugh classification and the benefit of ribavirin in addition to DAAs. Moreover, we could not directly compare the outcomes between DAA-treated patients who achieved SVR and those who did not because the available data were insufficient to explore. However, the impact of DAA-mediated viral elimination on liver-related and non-liver-related mortality in patients with HCV-related decompensated cirrhosis was reported in a propensity score-matching study between the DAA group who achieved SVR and those who did not (13). Further research is needed to investigate the benefits of SVR in patients with HCV-related decompensated cirrhosis treated with DAAs. Lastly, the study by Dellay et al. (24) was conducted in decompensated HCV cirrhosis patients who were listed for liver transplantation while the study was retrospectively compared between patients who received and not received DAA treatment which meant that immortal time bias could occur but we couldn’t exclude this study because population at baseline were matched with our inclusion criteria.

## 5. Conclusion

Direct-acting antivirals treatment has been demonstrated to be effective in increasing patient survival and HCC-free survival, and potentially reducing the development of HCC in individuals with HCV-related decompensated cirrhosis. Additionally, it was significantly associated with improvements in MELD scores within 6 months of treatment.

## Data availability statement

The original contributions presented in the study are included in the article/supplementary material, further inquiries can be directed to the corresponding author.

## Author contributions

TJ: Conceptualization, Data curation, Formal analysis, Funding acquisition, Investigation, Methodology, Visualization, Writing – original draft, Writing – review and editing. AC: Conceptualization, Formal analysis, Validation, Writing – review and editing. PI: Conceptualization, Validation, Writing – review and editing. ATa: Conceptualization, Data curation, Formal analysis, Validation, Writing – review and editing. AT: Writing – review and editing. AS: Conceptualization, Data curation, Supervision, Validation, Writing – review and editing, Funding acquisition, Visualization.
